# A 10-Year Cardiovascular Risk in Adults with Different Levels of Spiritual Health: Tehran Lipid and Glucose Study

**DOI:** 10.5334/gh.1169

**Published:** 2023-01-23

**Authors:** Parisa Amiri, Fataneh Ghadirian, Parnian Parvin, Leila Cheraghi, Davood Khalili, Shahram Alamdari, Fereidoun Azizi

**Affiliations:** 1 Research Center for Social Determinant of Health, Research Institute for Endocrine Sciences, Shahid Beheshti University of Medical Sciences, Tehran, Iran; 2 Department of Psychiatric Nursing and Management, School of Nursing and Midwifery, Shahid Beheshti University of Medical Sciences, Tehran, Iran; 3 Department of Epidemiology and Biostatics, Research Institute for Endocrine Sciences, Shahid Beheshti University of Medical Sciences, Tehran, Iran; 4 Prevention of Metabolic Disorders Research Center, Research Institute for Endocrine Sciences, Shahid Beheshti University of Medical, Tehran, Iran; 5 Obesity Research Center, Research Institute for Endocrine Sciences, Shahid Beheshti University of Medical Sciences, Tehran, Iran; 6 Endocrine Research Center, Research Institute for Endocrine Sciences, Shahid Beheshti University of Medical Sciences, Tehran, Iran

**Keywords:** spiritual experience, ACC-AHA risk score, gender, lifespan

## Abstract

**Background:**

Previous studies have shown that spiritual experience may reduce cardiovascular disease (CVDs). However, little is known about the relationship between spiritual health and the gender-specific risk of CVDs in communities with different cultures.

**Methods:**

A total of 3249 individuals (53.7% female, 75.0% middle-aged) participated in the Tehran Lipid and Glucose Study (TLGS) from 2015 to 2017 were included. Based on the ACC/AHA pooled cohort equation, CVD risk over ten years was examined. Spiritual health was measured using a developed tool for measuring spiritual health in Muslim populations (SHIMA-48). Linear regression models were used to assess the association between spiritual health and ACC/AHA risk scores. The natural logarithm scale was calculated to consider the normal distribution hypothesis of the regression model.

**Results:**

The current results suggest a slight but significant increase in the mean of spiritual health in women compared to men in both cognitive/emotional and behavioral dimensions (P < 0.001). In both sexes, a higher prevalence of smoking was observed in participants with lower levels of spiritual health (P < 0.004). In men, compared to those with a low level of spiritual health (the first tertile), the logarithm of the ACC-AHA risk score was reduced by 0.11 (P = 0.004) and 0.18 (P < 0.001) for those in the second and third tertiles of spiritual health, respectively. This result may be attributed to higher cigarette smoking among the latter group. Similar results were not observed in women.

**Conclusions:**

Current results indicate a gender-specific association between spiritual health and cardiovascular disease risk. Our findings imply that promoting spiritual health can be considered an effective strategy in future preventive interventions, primarily by controlling the desire to smoke in men.

## Introduction

Cardiovascular diseases (CVDs) are the leading cause of global mortality and morbidity [1]. In Iran, CVDs are responsible for 46% of deaths and 20–23% of the disease burden [2]. Some studies believe in a central and bidirectional relationship between cardiovascular health and spiritual experience [3,4]. Spirituality is a personalized phenomenon that naturally happens while seeking the meaning of life and connection with a superior power [5]. As the fourth dimension of health, as suggested by the world health organization (WHO), spiritual health is considered a dimension of health and not merely an influencing factor [6]. In recent decades, spiritual health has received much interest in health assessments, and well-being is believed to be the outcome of mental, physical, and spiritual health [7].

More detailed research on the mechanism of the effect of spiritual experience on cardiovascular health emphasized the positive relationship between cardiac function and spiritual well-being [3,8]. Spiritual health makes the parasympathetic overcome the autonomic nervous system [9]. Increased parasympathetic activity can lead to bradycardia, lower blood pressure, and decreased risk of CVDs by increasing acetylcholine [9,10]. In addition, spiritual health can improve cardiovascular functions by positively affecting hormonal, immune, and nervous mechanisms [5]. However, more evidence indicates that spiritual beliefs might predict the elevated CVD risk, which could be influenced by sex. Michgelsen et al. revealed that religious men in Europe had a lower 10-year CVD risk than their non-religious counterparts, while religious women in Ghana appear to have increased CVD risk [11]. Despite the proven gender differences regarding cardiovascular risk factors, including hypertension and overweight, and individual differences in psycho-spiritual responses [12], there is limited gender-specific evidence indicating an interaction between spiritual health and CVD risk.

Like many societies with an age transition, the Iranian elderly population is also projected to grow much more quickly in the following decades [13]. Iran has a high prevalence and mortality rate due to CVDs; twice in men than women [14], and the burden of CVD will double in 2025 compared to 2005 [15,16,17]. On the other hand, As a cultural element, religious beliefs and related rituals play an important role in shaping people’s lifestyle and spiritual health [18]. Consistent with the results of other countries, in Iran, increasing spiritual well-being is associated with a reduced risk of CVDs; this data can be used as an effective preventive strategy [19,20]. However, sex-specific data on future CVD risks are still unclear. Given that over 95% of Iran›s population are Muslims, and the intense entanglements of spiritual health and religion [21], examining the relationship between spiritual health and the risk of CVD using an accurate and culturally tailored tool can yield reliable results. Therefore, using the ACC-AHA risk score, validated criteria for the Iranian population [22], the present study aimed to assess the association between spiritual health status and a 10-year CVD risk score in men and women who participated in the last follow-up examination of the TLGS.

## Methods

### Study design and participants

This cross-sectional study was conducted in the context of the Tehran Lipid and Glucose Study (TLGS), a large-scale, population-based, prospective study initiated in 1999, aimed to monitor non-communicable diseases (NCDs) risk factors and consequences in an expected duration of at least 20 years. After the baseline assessment, ongoing follow-up examinations were conducted at three-year intervals. Participants were 15005 individuals aged ≥3 years, residing in district 13 of Tehran. All measurements were performed at the Lipid and Glucose testing unit, a community-based research site in the research area, and under the Research Institute of Endocrine Sciences of Shahid Beheshti University of Medical Sciences (SBMU) supervision. More details of the TGLS study were previously published [23].

**Figure 1:**
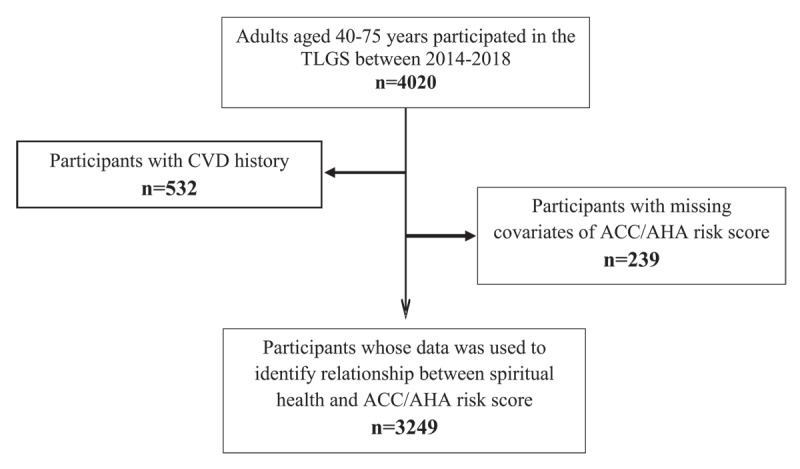
The sampling flowchart.

The current study recruited sociodemographic, behavioral, and cardio-metabolic data for 4020 adults aged 40–75 years with available spiritual health assessments who participated in the last follow-up of the TLGS between 2014–2017. After excluding those with CVD history (n = 532) and those with missing data on ACC/AHA risk score covariates (n = 239), 3249 eligible participants were recruited for the current analysis (Figure 1). Data on participants’ characteristics were obtained from the TLGS data bank. Spiritual health status was assessed using a developed questionnaire culturally tailored for the Iranian population completed by trained interviewers [24]. Marital status was categorized as married and unmarried. Furthermore, education level was classified as primary, secondary, and higher. Employment status was considered as unemployed and employed.

### Spiritual Health Inventory in Muslim Adults (SHIMA-48)

Spiritual health inventory in Muslim adults (SHIMA-48) is a developed instrument culturally tailored for Iranian adults and other communities with primarily Muslim populations. It contains three main themes, including 1) insight, 2) tendency, and 3) behaviors. The first two concepts are defined as a cognitive-emotional component. Each theme encompasses three categories: relation to God, self, and the environment. The final 48 items were rated on a 5-point Likert scale anchored at 1 to 5 (‘1 = Strongly disagree to 5 = Strongly agree for insight and tendency’ subscales, and ‘1 = Always to 5 = Never’ for behavioral subscale). The scores ranged from 0 to 100, with higher scores indicating better spiritual health.

The psychometric properties of the SHIMA-48 were assessed using face, content, and construct validity methods. All questionnaire items were easy to read and understand for a sample of 30 individuals, and the face validity of the questionnaire has been confirmed. In addition, a panel of experts confirmed qualitative and quantitative content validity (the mean CVR and CVI were 0.80 and 0.85, respectively) of 48 items. Exploratory factor analysis suggested a six-factor model in the structure of developed items, which was optimized in a two-factor model encompassing 1) cognitive/emotional and 2) behavioral constructs. The confirmatory factor analysis results indicated good fits for the proposed models. As measured by Cronbach’s alpha coefficients, the internal consistency exceeded the minimum reliability standard of 0.70 for all scales and subscales, all of which demonstrated satisfactory test-retest reliability over two weeks. The average time for subjects to complete the test was 12 minutes [24].

The investigational review board approved the study at the research institute for endocrine sciences of Shahid Beheshti University of Medical Sciences, Tehran, Iran. All participants provided written consent.

### Measurements and definitions

Systolic blood pressure (SBP) and diastolic blood pressure (DBP) were measured twice through a clinical examination, and the mean value was considered the participant’s blood pressure. After 12 to 14 h of overnight fasting, blood samples were collected to determine the fasting plasma glucose (FPG), two hours plasma glucose (2hPG), triglyceride (TG), and total cholesterol (TC) following a standardized protocol [25]. Type 2 diabetes (T2DM) was defined as fasting blood sugar FBS ≥ 126 mg/dl or two-hour post-load glucose ≥ 200 mg/dl or taking medication for diagnosed diabetes [26]. History of CVD/CHD includes those with any report of cardiovascular outcomes during their life. Cigarette smoking was defined as 1) current smokers, including occasional smokers and active daily smokers, and 2) never smokers, including those who never smoked. Physical activity was assessed using the Modifiable Activity Questionnaire (MAQ) [27] and defined as low, moderate, and high levels.

### Assessment of cardiovascular risk

The Pooled Risk Equations recommended by ACC/AHA for non-Hispanic white men and women were used to calculate the 10-year risk of hard CVD [28]. These equations included covariates of age, total cholesterol, HDL cholesterol, treated or untreated systolic blood pressure, history of diabetes, and current smoking status; the validity of these equations was previously evaluated in the TLGS [22].

### Statistical analysis

Normal continuous variables were expressed as mean ± sd, while frequency and percentages were reported for categorical variables. Socio-behavioral and cardio-metabolic characteristics among groups were compared using independent samples t-test, ANOVA, and Chi-square test as appropriate. The Benjamini and Hochberg procedure was used to test multiple independent hypotheses simultaneously and aimed to control the False Discovery Rate (FDR), defined as the expected ratio of false rejections to the number of total rejections [29]. In the current analysis, FDR was considered as 0.15. A multiple linear regression model assessed the association between risk scores and spiritual health. The normal distribution of calculated risk scores in men and women was checked using the graphical method and Kolmogorov-Smirnov Test. Since the lack of normal distribution of the dependent variable (risk score), the corresponding values were transformed into the natural logarithm scale. Restricted Cubic spline regression analysis was conducted to assess the linearity of the association between spiritual health and logarithm of risk scores and found a nonlinear relationship (p < 0.001). So, the association between spiritual health and the logarithm risk scale was not monotone (Figure 1-Appendix). Hence, we decided to consider different levels of spiritual health according to its tertiles in the current analysis. The spiritual health group was defined based on the tertiles of its distribution as first, second, and third for men and women. The estimated regression coefficient represents the difference in the logarithm scale of ACC-AHA risk scores in the second and third tertile spiritual health scores compared to the first tertile group. The model was adjusted for confounding sociodemographic variables, including age, employment, education level, and BMI. All analysis was split according to sex and was performed using SPSS statistical software version 22 (SPSS Inc., Chicago, IL, USA) and Stata software version 13.

## Results

A total of 3249 (53.7% female) participants were recruited for the current analysis. The mean ± SD for age and spiritual health scores in the present population are 53.24 ± 8.82 and 90.30 ± 8.12, respectively. In addition, the correlation coefficient between age and spiritual health scores was 0.21 (p < 0.001).

### Participants’ characteristics based on gender

Compared to women, men were more educated, employed, and married. Men were more physically active than their female counterparts (21.9% vs. 11.3%). Compared with men, women were likelier to report a family history of CVDs, higher BMI, higher cholesterol, and higher rates of antihypertensive medication consumption. In contrast, high SBP, DBP, FBS, and active smoking were more frequent in men than women. In terms of spiritual health status, mean scores of cognitive/emotional and behavioral components were significantly higher for women than their male counterparts (P < 0.001) (Table 1).

**Table 1:** Socio-behavioral, cardio-metabolic and spiritual characteristics of participants by sex.


	MALE (n = 1341)	FEMALE (n = 1908)

**Age (years)**	53.58 ± 8.89	53.00 ± 8.76

**Education level**		

Primary	319 (23.8)	694 (36.4)

Secondary	604 (45.0)	823 (43.2)

Higher	418 (31.2)	390 (20.5)

**Occupational status**		

Unemployed	334 (24.9)	1647 (86.4)

Employed	1006 (75.1)	260 (13.6)

**Marital status**		

Unmarried	49 (3.7)	317 (16.6)

Married	1290 (96.3)	1588 (83.4)

**Physical activity**		

Low	520 (39.7)	475 (25.1)

Moderate	504 (38.4)	1207 (63.7)

High	287 (21.9)	214 (11.3)

**Family history of CVD**	22 (1.6)	63 (3.3)

**Smoking**	270 (20.1)	50 (2.6)

**BMI (Kg/m2)**	27.82 ± 4.31	29.77 ± 4.94

**CHOL (mg/dl)**	189.06 ± 37.06	198.38 ± 39.35

**FBS (mg/dl)**	103.91 ± 32.13	99.84 ± 28.58

**Diabetes**	226 (16.9)	334 (17.5)

**SBP (mm Hg)**	120.29 ± 15.57	114.42 ± 16.74

**DBP (mm Hg)**	80.25 ± 9.42	75.87 ± 9.47

**Anti-hypertension drug**	165 (12.3)	341 (17.9)

Total spiritual health score*	89.18 (9.41)	91.09 (6.97)

Behavioral component	85.76 (11.11)	88.10 (8.77)

Cognitive/emotional component	91.63 (9.75)	93.23 (7.37)


Data are presented as numbers (%) or mean (SD). The total spiritual health score, as well as its behavioral and cognitive/emotional components, were significantly different between men and women (p < 0.001).

### Risk distribution based on gender

**Figure 2:**
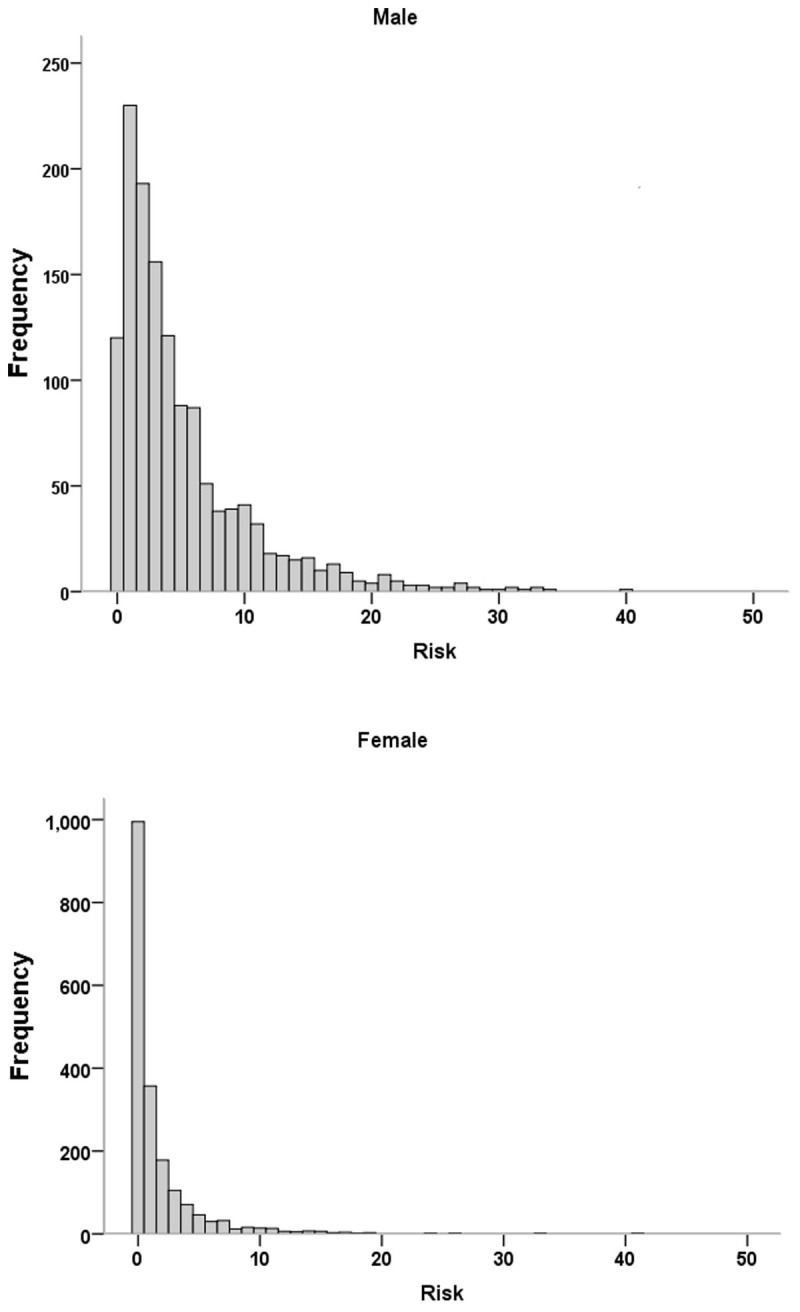
Histograms of the ACC-AHA risk score in males and females.

**Figure 3:**
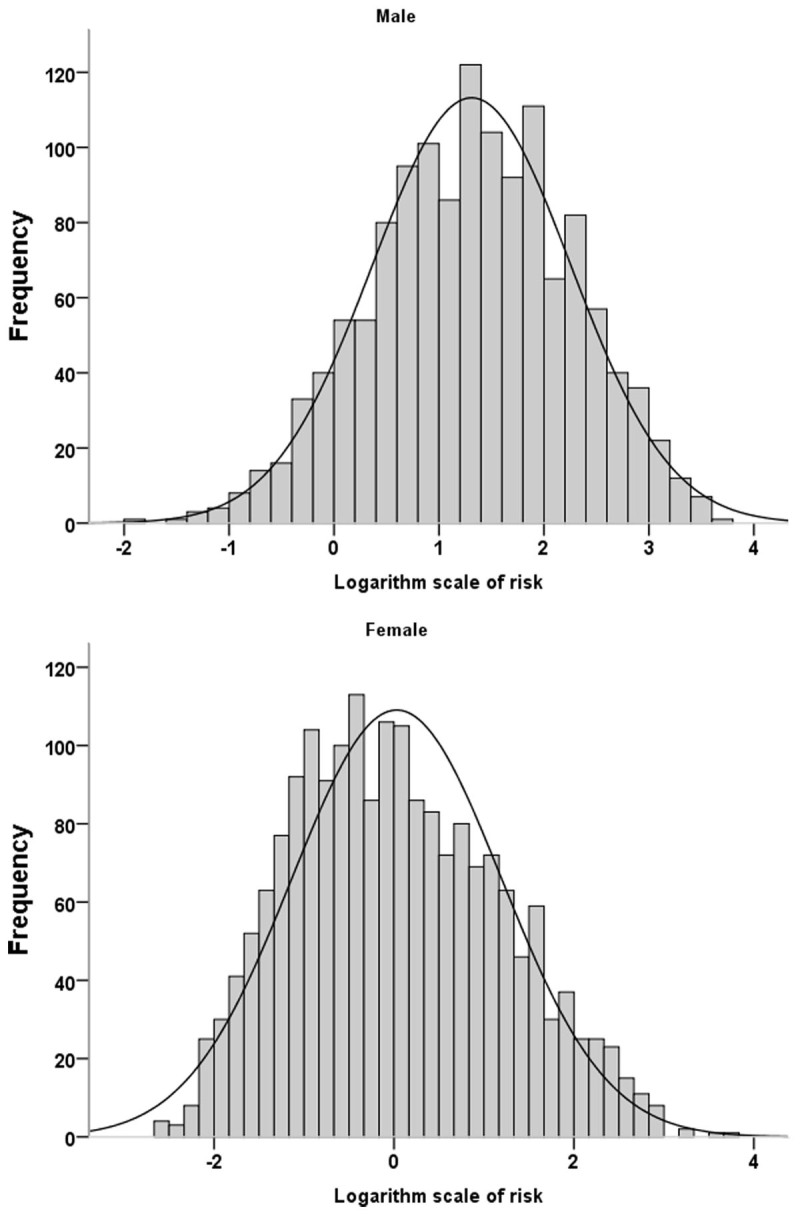
Histograms of logarithm scale of the ACC-AHA risk score in males and females.

Accordingly, the 10-year ACC/AHA risk score showed a skewness of risk distribution and differed between genders, with the highest in male participants (Figure 2). The median risk and interquartile range in men and women were 3.8 (1.9–7.3) and 0.9 (0.4–2.4), respectively. The log-transformed data follows a normal or near-normal distribution for both sexes (Figure 3).

### Association between participants’ characteristics and spiritual health status

Comparing participants in different spiritual health levels, our results indicated significant associations between spiritual health scores with age, education, and smoking in both sexes. Higher levels of spiritual health were significantly associated with lowered cholesterol in men and increased systolic blood pressure in women (p < 0.004 based on the Benjamin-Hochberg procedure) (Table 2).

**Table 2:** Socio-behavioral and cardio-metabolic characteristics based on the spiritual health tertiles in participants.


	MALE				FEMALE			
	
	1^ST^ TERTILE	2^ND^ TERTILE	3^RD^ TERTILE	p-VALUE	1^ST^ TERTILE	2^ND^ TERTILE	3^RD^ TERTILE	p-VALUE

**Age (years)**	50.53 ± 7.95	53.8 ± 8.58	56.45 ± 9.11	**3 × 10^–23^**	51.25 ± 8.02	53.04 ± 8.73	54.57 ± 9.16	**9 × 10^–11^**

**Education level**				**2 × 10^–6^**				**7 × 10^–9^**

Primary	91 (20.1)	84 (19.0)	144 (32.2)		165 (27.3)	237 (36.5)	292 (44.7)	

Secondary	196 (43.3)	220 (49.9)	188 (42.1)		295 (48.8)	271 (41.7)	257 (39.4)	

Higher	166 (36.6)	137 (31.1)	115 (25.7)		144 (23.8)	142 (21.8)	104 (15.9)	

**Occupational status**				**4 × 10^–8^**				0.008

Unemployed	143 (32.1)	120 (27.2)	71 (15.7)		501 (82.9)	566 (87.1)	580 (88.8)	

Employed	382 (84.3)	321 (72.8)	303 (67.9)		103 (17.1)	84 (12.9)	73 (11.2)	

**Marital status**				0.246				0.724

Unmarried	22 (4.9)	13 (3.0)	14 (3.1)		103 (17.1)	102 (15.7)	112 (17.2)	

Married	501 (82.9)	548 (84.3)	539 (82.8)		431 (95.1)	426 (97.0)	433 (96.9)	

**Physical activity**				0.462				0.961

Low	176 (39.6)	170 (39.3)	174 (40.1)		150 (25.0)	150 (23.1)	175 (27.0)	

Moderate	384 (63.9)	428 (66.0)	395 (61.1)		170 (38.3)	172 (39.7)	162 (37.3)	

High	98 (22.1)	91 (21.0)	98 (22.6)		67 (11.1)	70 (10.8)	77 (11.9)	

**Family history of CVD**	8 (1.8)	5 (1.1)	9 (2.0)	0.568	22 (3.6)	16 (2.5)	25 (3.8)	0.326

**Smoking**	127 (28.0)	83 (18.8)	60 (13.4)	**2 × 10^–7^**	29 (4.8)	12 (1.8)	9 (1.4)	**2 × 10^–4^**

**BMI(Kg/m^2^)**	27.72 ± 4.4	27.88 ± 4.17	27.85 ± 4.35	0.847	29.56 ± 5.02	29.53 ± 4.77	30.21 ± 5.02	0.021

**CHOL(mg/dl)**	193.52 ± 36.85	188.21 ± 38.14	185.36 ± 35.77	**35 × 10^–4^**	199 ± 38	199.08 ± 39.69	197.11 ± 40.25	0.595

**FBS(mg/dl)**	101.96 ± 29.96	104.91 ± 34.11	104.9 ± 32.2	0.284	97.5 ± 26.84	99.44 ± 27.51	102.4 ± 30.93	0.011

**Diabetes**	61 (13.5)	83 (18.8)	82 (18.3)	0.060	89 (14.7)	110 (16.9)	135 (20.7)	0.019

**SBP(mm Hg)**	118.83 ± 14.82	119.88 ± 15.75	122.16 ± 15.97	0.005	112.48 ± 15.79	113.78 ± 16.16	116.86 ± 17.87	**2 × 10^–5^**

**DBP(mm Hg)**	80.04 ± 8.99	80.62 ± 10.12	80.09 ± 9.14	0.600	75.67 ± 9.6	75.9 ± 9.46	76.03 ± 9.37	0.790

**Anti-hypertension drug**	37 (8.2)	62 (14.1)	66 (14.8)	0.004	93 (15.4)	117 (18.0)	131 (20.1)	0.097


The 1^st^ and 3^rd^ tertiles represent the most minor and highest spiritual health scores, respectively. Data are presented as numbers (%) or mean (SD). Bold values were significant at p < 0.004 based on the Benjamin-Hochberg procedure.

### Association between ACC/AHA risk score and spiritual health status

Unadjusted distribution of ACC-AHA risk scores showed no difference among different spiritual health levels (Figure 2-Appendix). However, after adjusting for potential confounders, the results indicated a significant negative association between spiritual health level and the ACC-AHA risk score on the logarithm scale only in men. Compared to the first level of spiritual health, the logarithm of the ACC-AHA risk score was reduced by 0.11 (95% CI: –0.19, –0.04, p = 0.004) and 0.18 (95% CI: –0.26, –0.10, p < 0.001) for those in the second and third levels of spiritual health, respectively. No associations were observed between ACC-AHA risk score and spiritual health in women, even after adjusting for confounders (Table 3).

**Table 3:** The association between spiritual health scores and logarithm scale of ACC-AHA risk scores: Results of linear regression models according to sex.


		MALE		FEMALE	
	
		β (95% CI)	p-VALUE	β (95% CI)	p-VALUE

**Model 1**	**2^nd^ vs. 1^st^ tertile**	–0.10 (–0.18, 0.02)	0.015	–0.03 (–0.09, 0.04)	0.429

	**3^rd^ vs. 1^st^ tertile**	–0.16 (–0.24, –0.08)	<0.001	0.03 (–0.04, 0.09)	0.473

**Model 2**	**2^nd^ vs. 1^st^ tertile**	–0.11 (–0.19, –0.04)	0.004	–0.02 (–0.08, 0.05)	0.548

	**3^rd^ vs. 1^st^ tertile**	–0.18 (–0.26, –0.10)	<0.001	0.01 (-0.06, 0.07)	0.758


* β represents the difference in the logarithm scale of ACC-AHA risk scores at 2^nd^ and 3^rd^ tertiles of spiritual health scores compared to the 1^st^ tertile (the least spiritual score); CI: confidence interval. Model 1 is adjusted for age. Model 2 is adjusted for age, education level, occupation status, and BMI.

## Discussion

The current study aimed to determine the CVD risk identified by the ACC-AHA risk score in Iranian men and women with different spiritual health levels. Present results confirm the previous findings regarding the sex disparities in predicting 10-year cardiovascular risk indicating higher risk scores in men than women. In addition, our results suggest a slight but significant increase in the mean of spiritual health in women compared to men in both cognitive/emotional and behavioral dimensions. Our results showed that men with higher levels of spiritual health were less likely to experience future cardiovascular diseases than their counterparts with poor spiritual health. This result would be primarily attributed to higher cigarette smoking among the latter group. Similar results were not observed in women.

In agreement with previous studies in Iran and other countries, our study confirms a sex-specific CVD risk [30,31,32,33]. This result may be due to the different prevalence of cardiovascular risk factors such as blood pressure, cholesterol, hemoglobin A1c, weight status, and smoking, as well as biological and pathophysiological differences in the incidence of CVD in each sex [34]. Findings confirm the current results regarding the disparity between the above-mentioned cardio-metabolic factors in men and women. A body of evidence showed that the lower CVD risk in women could be attributed to a protective effect of sex steroid hormones, especially estrogen [35]. Also, lower mean blood pressure in women can be due to a more robust anti-inflammatory mechanism to limit high blood pressure [36]. These differences make Iranian males, especially in middle age and elderly, almost two times more susceptible to statin therapy than women [22]. In addition, smoking is highly related to sex, particularly in low/middle-income countries [37]. Existing data confirm higher smoking prevalence in Iranian men than women [38] and could justify the higher CVD risk score among male participants of the current study.

In the present study, women had higher spiritual health than men, consistent with previous studies conducted in Iran and other countries [39,40]. There is still considerable debate about the reasons for these gender differences. Related hypotheses focus mainly on biological differences [41,42] and gender roles and socialization patterns in women and men [43,44], leading to sex-specific spiritual beliefs. According to Hall, while women’s role is defined as nurturing, supporting, and ensuring the well-being of others, men’s role has traditionally been related to self-esteem and personal well-being [45]. Therefore, women have a stronger emotional inclination towards God and a greater desire for religious affiliation, while men are interested in God’s power, knowledge, and activity [46]. These differences do not remain at the level of beliefs and are even evident in the spiritual behaviors of men and women. Studies have shown that women participate more in spiritual activities than men, including church participation and prayers [39,47].

The current results showed that men with higher levels of spiritual health were less likely to experience CVD outcomes during the future ten years than their counterparts with poor spiritual health. Consistent with our results, another study confirmed a positive correlation between religiosity/spirituality and a favorable cardiovascular profile by improving preclinical cardiovascular health predictors [48]. In the present study, systolic blood pressure in women and cholesterol in men were not clinically crucial despite significant statistical differences among various spiritual health levels. McIntash et al. believed that there is an indirect path between spiritual health and biological factors via unhealthy behaviors [49]. Accordingly, a Christian religious group has revealed the association between spiritual/religious life and positive and healthy behaviors such as smoking cessation and lowering cholesterol levels in both sexes [48]. In an eastern society like Iran with strong religious beliefs in prohibiting smoking, the recent conclusion that less smoking can be the main determining factor in reducing cardiovascular risk in men with higher levels of spiritual health seems quite logical. However, despite the significant reduction in smoking, there was no similar effect in reducing the risk of the mentioned diseases in women with higher levels of spiritual health. This gender difference could be due to the under-reporting of smoking in women due to cultural taboos or much lower risk of cardiovascular diseases in females than males.

Our study is one of the first to investigate a 10-year sex-specific risk of CVD in a Middle-Eastern population with a different cultural and religious context of western communities. Using SHIMA-48 as a culturally-tailored tool to assess the study’s spiritual health improves the results’ accuracy. In addition, due to possible differences between spiritual experiences in men and women, the current results could fill the literature gap regarding sex-specific associational mechanisms. A large community-based sample of men and women, the standardized assessment of biochemical variables and CVD risk factors, and the adjustment for crucial confounding variables are among the strengths of our analysis. However, our study had some limitations. Examining spiritual health in a religious community could lead to participants’ over-reporting, reducing the power of differentiation of various levels of spiritual health and increasing the tool’s ceiling effect. In addition, the cross-sectional nature of the current study limits us from investigating the causal relationships between study variables. Finally, restricting the sample to a specific urban region reduces generalizing the results.

The current results indicate a lower risk of CVD in men with higher spiritual health during the next ten years. Spiritual health plays a preventive role in cardiovascular risk by reducing men’s smoking tendencies. A finding was not observed in women, probably due to Iran’s different social norms and cultures. More sex-specific studies from culturally diverse populations must confirm and further elaborate on these findings.

## Data Accessibility Statement

All relevant data are within the paper and its Supporting Information files. The identified subject data may be available from the corresponding author on reasonable request for researchers who meet the criteria for access to confidential data.

## Additional File

The additional file for this article can be found as follows:

10.5334/gh.1169.s1 Supplementary File. Appendix Figures 1 and 2.
