# Supplementary material for: A 10-Year Cardiovascular Risk in Adults with Different Levels of Spiritual Health: Tehran Lipid and Glucose Study
Source: Glob Heart. 2023 Jan 23;18(1):1. doi: 10.5334/gh.1169 (PMC9881436; doi:10.5334/gh.1169)
Supplement: Supplementary File. — Appendix Figures 1 and 2. [file gh-18-1-1169-s1.pdf]

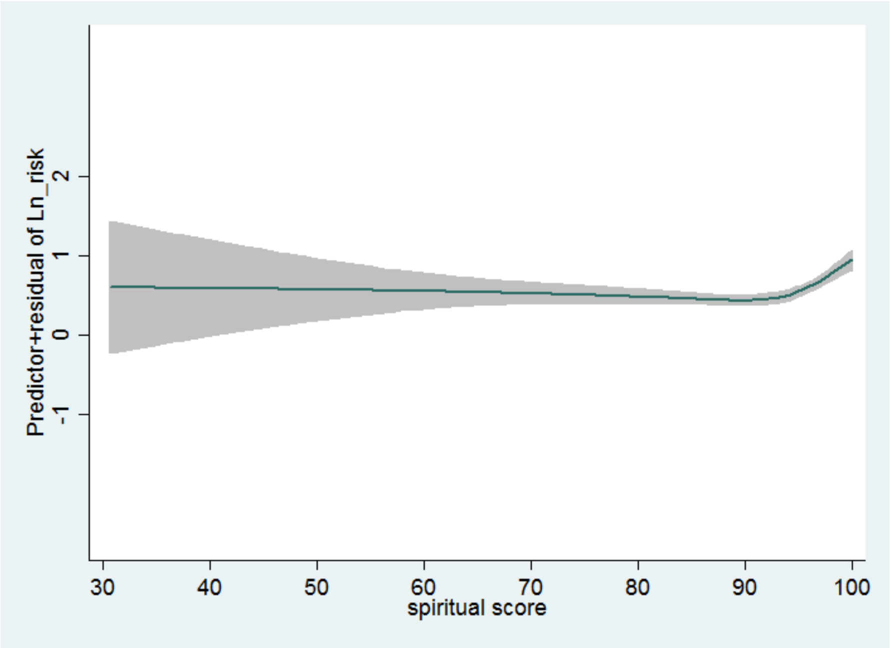

Figure 1-Appendix. The restricted cubic spline plot demonstrates the association between spiritual health and the logarithm of the ACC/AHA risk score.

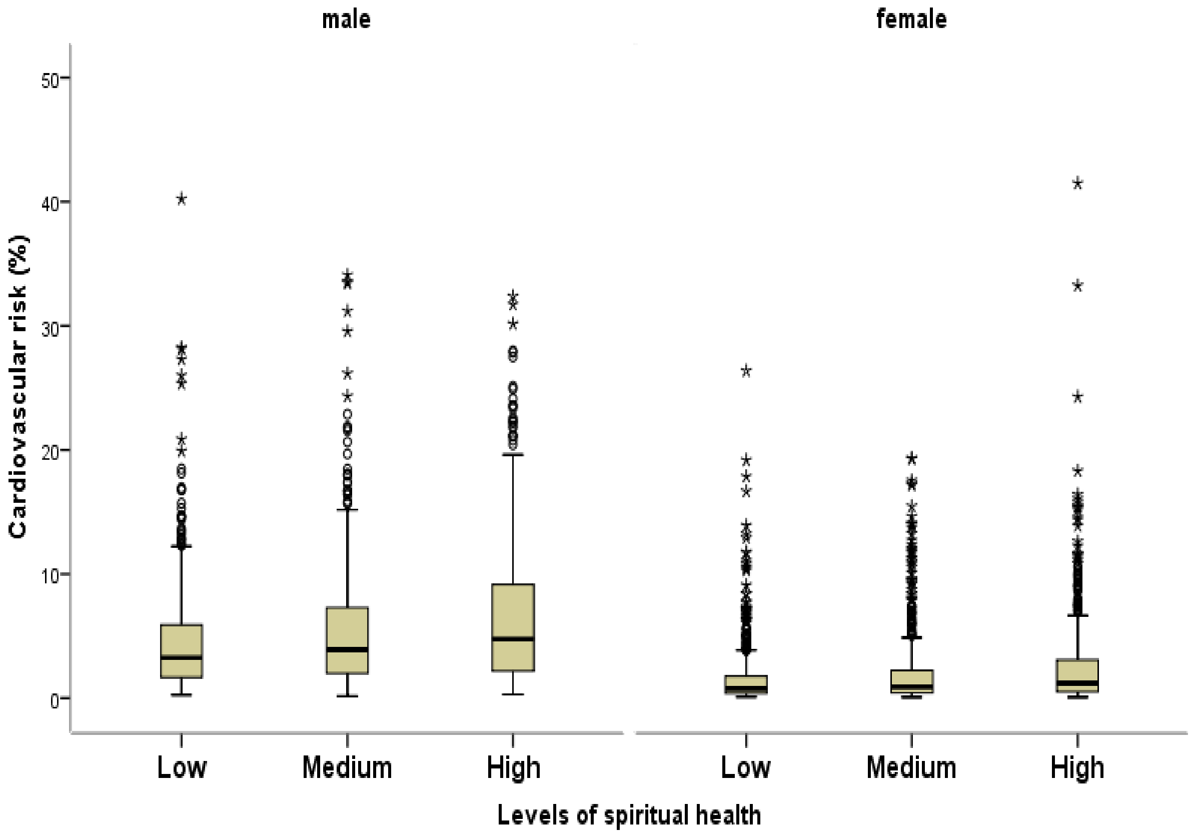

Figure 2-Appendix. Box plots of the ACC-AHA risk score in different spiritual health statuses according to sex.
